# Essential interventions: implementation strategies and proposed packages of care

**DOI:** 10.1186/1742-4755-11-S1-S5

**Published:** 2014-08-21

**Authors:** Zohra S Lassi, Rohail Kumar, Tarab Mansoor, Rehana A Salam, Jai K Das, Zulfiqar A Bhutta

**Affiliations:** 1 Division of Women and Child Health, Aga Khan University, Karachi, Pakistan

**Keywords:** Reproductive, maternal, newborn, child health

## Abstract

In an effort to accelerate progress towards achieving Millennium Development Goal (MDG) 4 and 5, provision of essential reproductive, maternal, newborn and child health (RMNCH) interventions is being considered. Not only should a state-of-the-art approach be taken for services delivered to the mother, neonate and to the child, but services must also be deployed across the household to hospital continuum of care approach and in the form of packages. The paper proposed several packages for improved maternal, newborn and child health that can be delivered across RMNCH continuum of care. These packages include: supportive care package for women to promote awareness related to healthy pre-pregnancy and pregnancy interventions; nutritional support package for mother to improve supplementation of essential nutrients and micronutrients; antenatal care package to detect, treat and manage infectious and noninfectious diseases and promote immunization; high risk care package to manage preeclampsia and eclampsia in pregnancy; childbirth package to promote support during labor and importance of skilled birth attendance during labor; essential newborn care package to support healthy newborn care practices; and child health care package to prevent and manage infections. This paper further discussed the implementation strategies for employing these interventions at scale.

## Essential interventions for maternal, neonatal and child health

Considering the interdependent relationship between maternal, neonatal and child health (MNCH), approaching essential interventions that have an impact on MNCH services has great potential for accelerating progress towards Millennium Development Goals (MDG) 4 and 5. The integration of these interventions needs to span not only in time period (pre-pregnancy, pregnancy, childbirth, and the postnatal period) but also across the levels of care (community, primary and referral). The delivery of these essential interventions can have profound translational and intergenerational impacts with important implications for the long-term well-being of mother, newborns and children. This can also promote greater efficiency by maximizing synergies and avoiding duplication of services that are less efficient if delivered individually. For example, the use of community based intervention packages have been shown to reduce maternal mortality, perinatal mortality, stillbirths and neonatal mortality [1]. Similarly, nutritional interventions for women prior to conception, such as folic acid supplementation, have long-term health benefits for the woman and can also promote healthier development of the fetus [2].

Pregnancy itself also represents a key window of opportunity for synergistic impact. For example, vaccination of the mother against tetanus during pregnancy can prevent neonatal tetanus and death associated with neonatal tetanus. During the most high-risk stage, i.e. delivery, these interventions is crucial and can have a profound impact on reducing maternal and neonatal mortalities. Skilled birth attendance can avert or manage the most common causes of mortality for both mothers and newborns, namely hemorrhage and complications arising from prematurity/low birth weight (LBW), respectively. A skilled birth attendant with the proper training and equipment can both manage excessive bleeding of the mother and also provide neonatal resuscitation. Further along the continuum of MNCH care, services during the postnatal period, such as early breastfeeding initiation, vaccinations, postnatal visits, and home based care can together have a profound impact on bolstering the health of the mother and neonate.

This paper summarizes essential interventions (table 1) which have been shown to have an impact on improving MNCH outcomes and could be used by both clinicians and policy makers in low and middle income countries (LMICs) programs. The paper further proposes packages of evidence-based effective interventions and strategies for delivery for such packages in varying contexts. Some interventions require technical expertise (e.g. management of eclampsia), whereas others can be provided at the community level (e.g. counseling for immediate and exclusive breastfeeding). Building on the evidence reviewed in this paper, the following essential interventions (refer to the link: http://www.who.int/pmnch/topics/part_publications/essential_interventions_18_01_2012.pdf) suggest a way forward in integrating and packaging MNCH across the continuum of care.

**Table 1:** Essential reproductive, maternal, newborn and child health interventions

Priority interventions	Level of care	Health care cadre	Key commodities
**Preconception/periconceptual interventions**			

Family planning [46,47-49]	Community Primary Referral	All	• Barrier methods (male and female • Condoms, diaphragm, gels, foams) • Oral contraceptives (progestin only and combined) • Emergency contraceptives and hormonal injections
	
	Primary Referral	Professional health workers	• All of the above plus implants • Long acting reversiblecontraceptives (implants) • Intrauterine devices • Surgical contraception

Prevention and management of sexually transmitted infections (STIs), including HIV for prevention of mother to child transmission (PMTCT) of HIV and syphilis [89,90]	Community Primary Referral	All	• Materials for counselling • Condoms (male and female) • Antibiotics in line with essential medicine guidelines
	
	Primary Referral	Professional health workers	• Materials for counselling • Condoms (male and female) • Antibiotics in line with essential medicine guidelines • Laboratory test kits for STI/HIV • Anti-retroviral medicines (refer to the essential list of medicines)

Folic acid fortification and/or supplementation to prevent neural tube defects [17-19]	Community Primary Referral	All	• Folic acid fortification of staple food e.g. flour • Folic acid tablets

Antenatal Care [91,92,93] Essential package	Primary Referral	Professional health workers	• Fetal stethoscope • Scale • Sphygmomanometer • Haemoglobinometer

Iron and folic acid supplementation during pregnancy [20,21,94]	Primary Referral Community	All	• Iron and folic acid

Tetanus immunization in pregnancy for preventing neonatal tetanus [13,14]	Primary Referral Community	All	• Vaccine (TT vaccine)

**Prevention and management of malaria in pregnancy** a) Prophylactic antimalarial for preventing malaria in pregnancy [8,9] b) Provision and promotion of use of insecticide treated nets for preventing malaria in pregnancy [11,95-97]	Primary Referral Community	All	a) Antimalarial drugs according to the situation/context b) Insecticide treated nets

Interventions for smoking cessation during pregnancy for improving birth outcomes [36,37]	Community Primary Referral	All	• Materials for individual and group counselling and behavioural change interventions on smoking cessation

Screening and treatment of syphilis [56,98]	Primary Referral	Professional Health Workers	• Onsite tests and laboratory equipment • Penicillin • Counselling material

Prevention and management of HIV and prevention of mother to child transmission in pregnancy [3-6]	Community Primary Referral	All	• HIV test kits • Antiretroviral drugs • Cotrimoxazole

**Prevention and management of hypertension in pregnancy** a) Calcium supplementation in pregnancy [22,23,24] b) Low-dose Aspirin for the prevention of pre-eclampsia in high risk women [24,57,58] c) Use of antihypertensive drugs for treating severe hypertension in pregnancy [59,60] d) Prevention and treatment of Eclampsia [24,61-64]	a) Community Primary Referral b) Primary Referral c) Primary Referral d) Primary Referral	a) All b) Professional health workers c) Professional health workers d) Professional health workers	a) Calcium b) Low dose aspirin c) Methyldopa, hydralazine, nifedipine d) Magnesium sulphate (Injection)

Reduce malpresentation at term using external cephalic version (>36 weeks) [72-77]	Referral	Professional health workers	• Stethoscope

Management of prelabour rupture of membranes and preterm labour: A) Induction of labour for management of prelabour rupture of membranes at term [51]	Referral	Professional health workers	• Uterotonic (oxytocin and/or misoprostol) • Partograph • Stethoscope

B) Antibiotics for management of preterm rupture of membranes [99,100]	Primary Referral	Professional health workers	• Antibiotic (erythromycin)

C) Corticosteroids for prevention of neonatal respiratory distress syndrome [65,66,67].	Primary Referral	Professional health workers	• Corticosteroids (betamethasone, dexamethasone)

**Management of unintended pregnancy: **[68] a) Availability and provision of safe abortion when indicated and legally permitted b) Provision of post abortion care	Primary Referral	Professional Health Workers	• Materials for counselling, health education and health promotion • Medications for induced abortion (mifepristone, misoprostol) • Vacuum aspiration equipment • Uterotonics (misoprostol, oxytocin) • Antibiotics in line with essential medicine guidelines • Surgical procedures when required

**Childbirth**			

Social support during childbirth [29]	Community Primary Referral	All	

Prophylactic antibiotic for caesarean-section [71]	Referral	Professional health workers	• Antibiotics (ampicillin or cefazolin)

Prevention of postpartum haemorrhage A) Prophylactic uterotonic to prevent postpartum haemorrhage [34,101]	Community Primary Referral	All	• Uterotonics (oxytocin, misoprostol)

B) Active management of third stage of labour to prevent postpartum haemorrhage [30,31-33].	Primary Referral	Professional health workers	• Uterotonics (oxytocin, ergometrine)

Induction of labour for prolonged pregnancy [78,79]	Referral	Professional health workers	• Uterotonics (oxytocin, misoprostol)

Caesarean section for absolute maternal indication (e.g. obstructed labour and central placenta previa)	Referral	Professional health workers	• Surgical environment

**Management of post-partum haemorrhage** e.g.: a) Uterine massage [102] b) Uterotonics [35,103] c) Manual removal of placenta (only by professional health workers)	Community Primary Referral	Community health workers (a+b) and referral.	• Uterotonics (oxytocin, ergometrine, misoprostol) • IV fluids • Blood transfusion • Surgical facilities

**Postnatal – mothers**			

Advice and provision of family planning [104]	Community Primary Referral	All	• Barrier methods (male and female condoms, diaphragm, gels, foams) • Oral contraceptives (progestin only and combined) • Long acting reversible contraceptives (implants and injectable contraceptives) • Intrauterine devices • Surgical contraception • Emergency contraception

Prevent, measure and treat maternal anaemia [105]	Referral	Professional health workers	• Ferrous salt (liquid or tablet) • Ferrous salt + folic acid (tablet) • Folic acid (tablet) • Nutritional supplements • Hydroxycobalamine (injection) • Lab tests • Blood products

Detection and management of postpartum sepsis [106]	Referral	Professional health workers	• Antibiotics (ampilcillin, gentamicin, metronidazole)

Screening and initiation or continuation of antiretroviral therapy for HIV [53]	Primary Referral	Professional	• Antiretroviral medicines

**Birth and postnatal – newborn**			

**Immediate essential newborn care (at the time of birth)**			

Promotion and provision of thermal care for all newborns to prevent hypothermia (immediate drying, warming, skin to skin, delayed bathing) [38]	Community Primary Referral	All	• Materials for counselling, health education and health promotion

Promotion and support for early initiation and exclusive breastfeeding (within the first hour) [1,42-44,107,108].	Community Primary Referral	All	• Materials for counselling, health education and health promotion

Promotion and provision of hygienic cord and skin care [16,109,110]	Community Primary Referral	All	• Cord clamp and scissors • Clean birth kit for health facilities

Neonatal resuscitation with bag and mask for babies who do not breath at birth [39-41].	Community Primary Referral	Professional health workers	• Training aids and devices to maintain competencies • Newborn resuscitation device (Ambu Bag, bag-mask and suction device)

Newborn immunization	Primary Referral	Professional health workers	• Vaccines, syringes, safety boxes, cold chain equipment

**Neonatal infection management**			

Presumptive antibiotic therapy for the newborns at risk of bacterial infection [111]	Referral	Professional health workers	• Antibiotics (ampicillin and gentamycin or penicillin)

Case management of neonatal sepsis, meningitis and pneumonia [112-115]	Community Primary Referral	All (Community refer)	• Materials for counselling, health education and health promotion • Thermometer / digital thermometer • Timer • Blood sugar sticks (disposable) • Nasogastric tube • Antibiotics (oral and injectable)

Kangaroo mother care for preterm and for < 2000g babies [69,70]	Primary Referral	Professional health workers	• Materials for counselling, health education and health promotion • Support Binder for KMC (KMC wrap) • Hat • Nasogastric tube

Extra support for feeding the small and preterm baby [116]	Primary Referral	Professional Health Workers	• Nasogastric tubes • Feeding cups • Breast pump • Syringe drivers • Blood sugar testing sticks • Materials for counselling

Prophylactic and therapeutic use of surfactant to prevent respiratory distress syndrome in pre-term babies [83-85]	Referral	Professional health workers	• Surfactant (await generics) • Oxygen supply/concentrator • Pulse oximeter

Continuous positive airway pressure (CPAP) to manage pre-term babies with respiratory distress syndrome [80-82]	Referral	Professional health workers	• Standard CPAP or bubble CPAP • Oxygen supply/concentrator • Pulse oximeter

Management of newborns with jaundice [117,118]	Primary Referral	Professional health workers	• Bilirubinometer • Phototherapy lamp • eye shade • IV fluids • Exchange transfusion kit

**Infancy and childhood**			

Promotion and support for exclusive breastfeeding for 6 months [119-121]	Referral Primary Community	All	• Materials for counselling, health education and health promotion, including individual and group counselling

**Promotion and support of continued breastfeeding and complementary feeding** a) Continued breastfeeding up to 2 years and beyond [122] b) Appropriate complementary feeding starting at 6 months [45,123-125]	Referral Primary Community	All	• Materials for counselling, health education and health promotion

**Prevention and management of childhood malaria** a) Provision and promotion of use of insecticide treated bed nets for children [11] b) Case management of childhood malaria [10,97,126,127]	Community Primary Referral	All	• Materials for counselling, health education and health promotion • Insecticide treated nets • Rapid diagnostic tests • Antimalarial drugs according to guidelines

Comprehensive care of children infected or exposed to HIV infection [52-55]	Referral Primary	Professional health workers	• Antiretroviral drugs • HIV test kits • Cotrimoxazole • Psychosocial support • Nutritional support

Promote and provide routine immunization plus H.influenzae, meningococcal, pneumococcal, and rotavirus vaccines [12,128-130]	Community Primary Referral	All	• Materials for counseling, health education and health promotion • Vaccines, syringes, safety boxes, cold chain equipment

Vitamin A supplementation from 6 months of age in Vitamin A deficient populations [131,132,133]	Community Primary Referral	All	• Vitamin A capsules • Material for counselling on Vitamin A rich foods

**Management of severe acute malnutrition: **[28,134] a) Without complications (All levels) b) With complications (Referral)	Community Primary Referral	All	**Community level** • Appropriate ready-to-use therapeutic foods • Micronutrient supplements • Vitamin A capsules **Health facility level** • Antibiotics • Therapeutic food formulations (F75/100)

**Case management of childhood pneumonia **[15,135-137] a) Vitamin A as part of treatment for measles-associated pneumonia for children above 6 months [25,26] b) Vitamin A as part of treatment for non-measles-associated pneumonia for children above 6 months [138,139,140]	Community Primary Referral	All	**Community level** • Respiratory rate timers • Vitamin A capsules **Health facility level** • Antibiotics **Referral level** • Oxygen for severe pneumonia • Pulse oximeter

**Case management of diarrhoea:** a) Acute watery diarrhoea [27,141-146] b) Dysentery [147,148,149]	Community Primary Referral	All	• Materials for counselling, health education and health promotion • Zinc (tablets / solution) • ORS • Appropriate antibiotics for dysentery according to guidelines

Case management of meningitis	Referral	Professional health workers	• Appropriate antibiotics • Supportive treatment

**Cross-cutting community strategies**			

Home visits across the continuum of care women’s groups [1,86,87,150,151]	Community	All	• Material for counselling, health education and health promotion

## Summary of essential interventions applicable at all levels: community, primary, and referral

### Infectious diseases and immunization

#### HIV and sexually transmitted infections (STI)

Mass treatment and behavioral interventions to increase awareness can non-significantly reduce the incidence of STIs and HIV [3-6]. Antiretroviral therapy (ARV) given in order to reduce the transmission of HIV showed that short courses of ARV drugs are effective in reducing transmission [7]. It was noted giving either the combination of zidovudine (ZDV) and lamivudine (3TC) to mothers during antenatal, intrapartum and postpartum period and to babies for a week after delivery or a single dose of nevirapine (NVP) given to mothers in labor and babies immediately after birth may be most effective in reducing the rates of vertical transmission [7].

#### Malaria

Antimalarial drugs reduce antenatal parasitemia when given to pregnant women in malarial endemic areas with greater benefit is seen in primi- and secundi-gravidas [8]. Sulfadoxine-pyrimethamine regimen is feasible, though their regular use may precipitate increased risk of resistance. It is also associated with lower incidence of LBW (35%) and all-cause child mortality (18%) [9]. The evidence showed increased protective efficacy of artemisinin-based combination therapies for treating uncomplicated malaria [10]. Effective case management of malaria including parenteral quinine for treating severe P. falciparum malaria also displayed protective impact on malaria mortality in children under five [11].

The use of insecticide treated nets (ITNs) is very effective in reducing perinatal morbidity and mortality [11]. It reduces peripheral and placental parasitemia, increases mean birth weight, and decreases the risk of fetal loss in the women in their first to fourth pregnancies. ITNs therefore should be an integral part of strategies to prevent malaria in pregnant women living especially in areas where malaria is endemic. There is strong evidence that ITNs use can reduce child mortality by 18% [11].There is a need for large-scale implementation of this intervention in malarial endemic areas.

#### Immunizations

Efforts are being made to maximize the coverage of immunization (EPI and EPI plus H influenza, pneumococcal conjugate vaccination, rotavirus) and the newer vaccines to avoid preventable deaths in women and children. Current evidence shows that rhesus rotavirus vaccines (particularly RRV-TV) and the human rotavirus vaccine 89-12 are efficacious in preventing rotavirus caused diarrhea and all-cause diarrhea [12]. Although, evidence about safety, or prevention of severe outcomes, is scarce and inconclusive. The current evidence also supports the implementation of tetanus immunization in pregnancy in communities with similar or higher levels of risk of neonatal tetanus [13,14].

#### Case management of neonatal and childhood infections

Case management of pneumonia could result in a 35% reduction in mortality from pneumonia in 0–5-year-old children [15]. On the basis of available evidences, it can be concluded that antibiotics have a clear role in reducing neonatal mortality as a result of meningitis, pneumonia and sepsis, in low income areas and can be effectively administered in homes via trained health workers.

#### Hygienic cord and skin care

Use of antiseptic chlorhexidine is associated with lower risk of bacterial colonization (with S. aureus, Streptococci and E. coli), neonatal mortality and omphalitis [16].

### Nutritional rehabilitation and micronutrient supplements

#### Folic acid

Folic acid fortification/supplementation to prevent neural tube defect (NTD) has a significant protective effect on NTDs, particularly in women who had a previous pregnancy affected by it (recurrent NTDs) [17-19]. Therefore, all women with a history of baby with NTDs should be advised and given folic acid to prevent its recurrence. Providing mothers during pregnancy with folic acid and iron also reduces the risk of being anemic near or at term.

#### Iron

Providing mothers during pregnancy with iron reduces the risk of being anemic near or at term [20,21]. These reviews suggested that newborn, whose mothers were provided with iron supplementation during the antenatal period, showed reduced risk of low birth weight and premature births.

#### Calcium

Calcium to prevent hypertension significantly reduces the risk of pre-eclampsia by 55% and by 64% in women with low dietary intake of calcium [22]. It also had a significant protective effect on maternal mortality and serious morbidity and preterm birth [22-24].

#### Vitamin A

Vitamin A supplementation as prevention and treatment of measles and pneumonia in children after six months of age reduces all-cause mortality and measles specific mortality [25,26]. Due to its beneficial effect on all-cause and diarrhea-specific mortality, routine vitamin A supplementation in this age group has thus been recommended.

#### Zinc

Therapeutic zinc supplementation has a beneficial effect on reducing duration and severity of acute and persistent diarrhea [27]. On the basis of these results, it is proposed that zinc supplementation should especially be applied to low income countries where the burden of diarrheal diseases is the highest. In contrast, treatment of pneumonia episodes with zinc is has shown no beneficial impacts on reducing pneumonia mortality.

#### Nutritional rehabilitation

Proper implementation of WHO guidelines for management of severe acute malnutrition (SAM) can decrease morality by 61% in hospital setting [28]. Children who were moderately or severely malnourished show better weight gain and higher recovery rates with ready-to-use therapeutic food than other management options such as F-100 or corn flour diet.

### Antenatal and birth care

#### Continuous social support

Continuous social support has shown significant clinical benefits for women and infants. Continuous support was more likely to have a spontaneous vaginal birth (8%), less likely to have intrapartum analgesia (10%), less likely to have a caesarean (21%) or instrumental vaginal birth (10%) [29]. Therefore, it is recommended that all women should receive continuous social support throughout labor and childbirth.

#### Intra and post-partum hemorrhage (PPH)

It was noted that active management is superior to expectant management in terms of reduction in the risk of maternal hemorrhage at time of birth [30-33]. However, significant results in the improved outcomes of blood loss and PPH could not be found when active management was employed. On the other hand, prophylactic oxytocin showed benefits in reducing blood loss >500ml and need for therapeutic oxytocics [34].

Misoprostol when compared with placebo was found to be effective in reducing severe PPH and blood transfusion [35]. In comparison with conventional injectable uterotonics, oral misoprostol was associated with higher risk of severe PPH and use of additional uterotonics, but with a trend to fewer blood transfusions.

#### Smoking cessation

Smoking cessations in pregnancy have shown to reduce preterm birth, low birth weight, and bring an increase in mean birth weight [36,37]. It is important that smoking cessation programs be implemented in all maternity care settings.

### Neonatal care and breast feeding

#### Hypothermia

Immediate thermal care is an important delivery care, leading to fatal morbidities and consequent mortality. Any intervention other than primary care designed for prevention of hypothermia, and applied within 10 minutes after birth in the delivery suite, may be beneficial in practice [38]. The interventions include: plastic wraps and bags, skin-to-skin contact, and transwarmer mattresses etc. These interventions keep infants warmer and lead to higher temperatures on admission to the NICU and to decreased incidence of hypothermia. To prevent the morbidity and mortality in preterm infants due to hypothermia, consideration should be given to using these interventions in the delivery suite.

#### Neonatal resuscitation

Evidence has shown that neonatal resuscitation with bag and mask has a vital role in saving newborn lives [39-41]. However, the feasibility of scaling up this approach is unclear. Facility based resuscitation training found reductions in intrapartum related neonatal mortality; while community based resuscitation training found reduction in all cause and asphyxia related neonatal mortality. No randomized, controlled trials were found to support or refute that the administration of epinephrine to the apparently stillborn or extremely bradycardic newborn infant reduces mortality and morbidity. Similarly, we found no randomized, controlled trials which addressed the issues of optimum dosage and route of administration of epinephrine.

#### Breast feeding and complementary feeding

Individual and group counseling to pregnant mothers showed an increase in rates of mothers exclusively breastfeeding in the neonatal period and at six months [42,43]. Also, group counseling showed better results of mothers who breastfeed in the neonatal period and at 6 months compared to those mothers who were individually counseled on the benefits of breastfeeding [44]. Current evidence suggests that interventions to promote complementary feeding have a positive impact on health outcomes of infants, including stunting and weight gain. These impacts are significant in both food-secure as well as insecure populations [45].

#### Family planning and contraception

Short inter-pregnancy interval (IPIs) is associated with a higher risk of preterm birth, low birth weight, fetal death and small for gestational age infants compared to IPIs of 18 - 23 months [46,47-49]. The risk is also high for these three adverse outcomes if the infant is conceived 60 months or more after a birth. Therefore family planning should be advised to plan pregnancies at appropriate intervals.

Postpartum education about contraception is also found to improve contraception use and fewer unplanned pregnancies [50]. The effectiveness of postpartum education about contraceptive use has not yet been established in randomized controlled trials. Such education may be effective in increasing the short-term use of contraception.

## Summary of essential interventions at primary and referral levels

### Infectious diseases

#### Chorioamnionitis

For preterm, premature rupture of membrane (PPROM), the use of antibiotics was associated with a significant reduction in chorioamnionitis, preterm birth and neonatal infections, which would support their routine clinical use in such cases [51]. It did not, however, significantly impact perinatal mortality and neonatal mortality.

#### HIV and STIs

A regimen combining triple ARV is most effective for preventing transmission of HIV from mothers to babies [7]. The risk of adverse events to both mother and baby appears low in the short-term but the optimal ARV combination and the optimal time to initiate this to maximize prevention efficacy without compromising the health of either mother or baby remains unclear.

There is strong evidence for HIV-infected women to exclusively breastfeed for 6 months, however, the decision on continued breastfeeding remains unclear and further evidence is required [52,53-55]. For children with HIV infections, the results of the Cochrane review on co-trimoxazole prophylaxis showed that the children who received the prophylactic treatment survived longer and had reduced number of days spent in hospital [52].

The review on antibiotics given for the management of syphilis did not have any trial that met the eligibility criteria. However, observational studies showed lower incidence of neonatal mortality by 82% and preterm delivery by 64% [56].

### Antenatal care

#### Hypertension, pre-eclampsia and eclampsia

The results show that when low dose aspirin are given during pregnancy, it lead to consistent reduction in the risk of preterm births (less than 34weeks), pre-eclampsia and other adverse effect of pregnancy [24,57,58]. Importantly for the fetus there is a significant reduction of 17% in mortality (fetal, neonatal, and infant). We would recommend that anti-platelet agents should be given to pregnant women at high risk of pre-eclampsia or those with gestational hypertension, since they lead to reduction of vast adverse outcomes of pregnancy both for the mother and newborn.

The use of anti-hypertensive causes a reduction in progression to severe hypertension [59,60]. The impact on eclampsia and perinatal/neonatal mortality was not significant; however, oral beta-blockers decrease the risk of severe hypertension by 63% and the need for additional antihypertensives by 54%.

Magnesium sulphate is an inexpensive drug and can be conveniently used in low income countries. The review indicates that this drug significantly reduces the progress to eclampsia when given to women with pre-eclampsia [24,61-64].

#### Use of corticosteroids

Corticosteroids to prevent RDS in newborn showed a significant 31% reduction in neonatal deaths (31%). There was also a significant 34%, 45%, and 46% reduction in the incidence of RDS, cerebroventricular hemorrhage and severe cerebroventricular hemorrhage, respectively [65-67].

#### Safe abortions

According to the WHO guidelines, safe abortion services, as provided by law, need to be available, provided by well-trained health personnel supported by policies, regulations and a health systems infrastructure, including equipment and supplies, so that women can have rapid access to these services [68].

#### Intra and postpartum care

It was noted that active management of third stage of labor to prevent postpartum hemorrhage is far superior to expectant management [30-33]. This was seen with significant results in the outcomes of blood loss, postpartum hemorrhage, severe postpartum hemorrhage, need for blood transfusion and postpartum anemia.

### Immediate and essential newborn care

#### Hypothermia

Evidence synthesized from a number of RCTs shows that Kangaroo Mother Care (KMC) has a large effect on mortality and is also effective in reducing morbidity [69,70]. This evidence is sufficient to recommend the routine use of KMC in facilities in babies <2000g at birth. The potential effect of KMC is greatest in low-income countries, where other options for care of preterm babies remain limited with few neonatal care facilities, mainly in distant referral hospitals and those that are often understaffed and ill-equipped.

## Summary of essential interventions at referral levels only

### Infectious diseases

For the prophylactic antibiotic for caesarean section, the results from Cochrane review concludes that the combination of clindamycin and an aminoglycoside (such as gentamicin) is appropriate for the treatment of endometritis, the most common cause of puerperal fever and sepsis. Prophylactic antibiotic administration in caesarean section was found to reduce endometritis and wound infections [71].

There are insufficient data from randomized controlled trials to guide clinical practice. A large randomized controlled trial is needed in asymptomatic term infants born to mothers with risk factors for infection in their babies, which compares the effect of prophylactic versus selective antibiotics on morbidity, mortality and costs.

### Intra and postpartum care

#### Breech presentation

For reducing mal-presentation at term with external cephalic version, there is sufficient evidence to conclude that external cephalic version at term reduces the chances of non-cephalic births and caesarean sections [72-77].

On the basis of current evidence, it can be concluded that a policy of planned caesarean section compared with planned vaginal birth, for singleton term breech presentation, is associated with a decrease in perinatal or neonatal death and/or neonatal morbidity. Although there is an associated perinatal morbidity, the most relevant outcome is the reduction in perinatal/neonatal death. This protective benefit outweighs the risks associated with caesarean section. Planned Caesarean section is therefore recommended for delivery of the baby with breech presentation compared to vaginal delivery.

#### Prolonged pregnancy

In the case of absence of a specific disorder, induction of labour can be proposed in patients between 41(+0) and 42(+6) weeks (grade B) [78,79]. The choice of prolongation beyond above 42(+0) weeks appears to involve an increase in fetal risk. This must be explained to the patient and balanced against the potential disadvantages of induction to enable an informed decision. This policy is associated with fewer deaths (69% perinatal deaths). There does not seem to be any increased risk of assisted vaginal or abdominal delivery. If the woman chooses to wait for spontaneous labor onset it would be prudent to have regular fetal monitoring as longitudinal epidemiological studies suggest increased risk of perinatal death by increasing gestational age.

### Small and sick newborn care

#### Mechanical ventilation for respiratory distress syndrome (RDS)

Noninvasive positive pressure ventilation (NIPPV) compared to Continuous Positive Airway Pressure (CPAP) to manage babies with RDS may be a useful method in preterm infants with apnea [80-82]. The review shows that there are more benefits in treating neonates with triggered ventilation compared to conventional ventilation. There was a reduction in both air leaks and duration of ventilation [80].

#### Surfactant use

Another important intervention pertaining to preterm survival is surfactant administration. Infants who underwent prophylactic administration of synthetic surfactant had a decreased risk of pneumothorax, pulmonary interstitial emphysema and neonatal mortality [83-85]. Also infants who were given synthetic surfactant showed an increased risk of developing patent ductusarteriosus and pulmonary hemorrhage. Results also show that multiple doses of surfactant as prevention or treatment resulted in reduction in the incidence of pneumothorax, mortality and necrotizing enterocolitis [83,84,85].

### Summary of cross cutting community strategies

The available data suggests that introduction of community based intervention packages can improve maternal and neonate survival [1,86,87]. The results from Cochrane review are very promising and showed impact on reducing stillbirths, perinatal mortality and neonatal mortality [1]. According to available evidence, it is recommended that home visits should be initiated as soon as possible after birth or after returning home from the facilities. A visit within the first 24 hours after birth is likely to be most effective in reducing newborn mortality. Additional visits on day 3 and, if possible, on day 7 can improve home care practices and identify danger signs or illness. Low birth weight babies need additional care to survive and stay healthy. This includes greater support for keeping them warm, initiating early and exclusive breastfeeding, and preventing infections.

### Proposed packages of care

The essential interventions identified in this paper can be bundled together and delivered in various packages to increase efficiency and provide a synergistic effect. These interventions can be bundled into the seven packages for delivery. In these seven packages of care, several means of delivery exist, and should be adopted in a context-specific manner. Critical to the success of delivering these packages, however, will be the support of several key players, including community health workers (CHWs) and professional health workers in first-level facilities as providers of care. For successful implementation of these interventions, these cadre of workers need to be trained. Funding should also be comprehensive and sustained to deliver these interventions across the household to hospital continuum of care in affordable and accessible manner.

**Figure 1:**
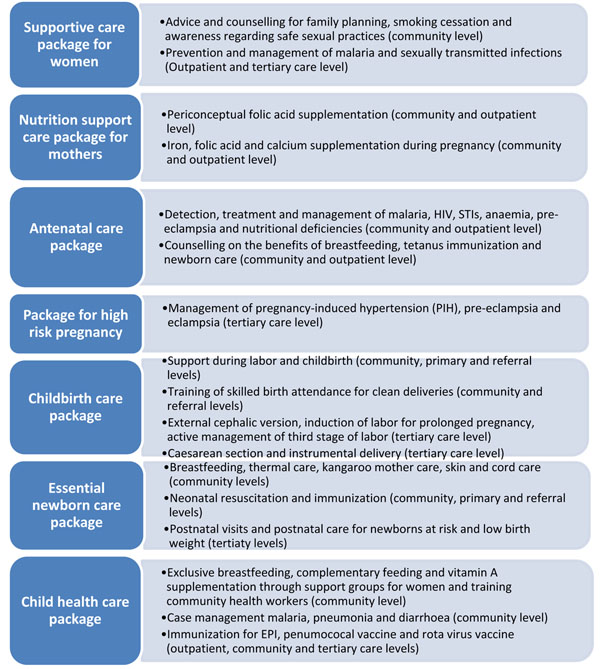
Packages for reproductive, maternal, newborn and child health care delivery systems

The interventions within the packages identified are, to a large extent, low-tech, low-cost, and have been implemented in an effective manner with strong impact. These packages should be implemented incrementally, with close coordination and strengthening of the existing health system beginning at the community and district level. Creation of these packages can also support in the rationalization of limited resources in a more effective and evidence-based manner. A similar package has been proposed earlier for maternal and newborn interconnected interventions [88]. Therefore, we have also incorporated interventions in the packages designed earlier to support their implementation [88]. The seven proposed packages are as follows and are summarized in figure 1

#### 1. Supportive care package for women

Supportive care packages for women are crucial for the wellbeing of both the mother and the child. This package supports the health of the mother and child during pregnancy and during the postpartum period through promoting healthy behaviors and emotions, including advice and counseling for family planning, smoking cessation, awareness regarding safe sexual practices and prevention and management of malaria which can be provided through community based support groups. These packages should be introduced at all levels of health care and must be readily available to all the women who belong to the reproductive health group.

To increase the reach of this particular intervention, CHWs or outreach workers must be trained to provide counselling at the community level. These packages should also exist at the basic health units and the rural health care centres at the primary level of health care. On the referral level, health care professionals should be trained to provide counselling and advice to those seeking it. The package should include important information to be dispensed to new mothers or those who plan to conceive. Women and their partners should be counselled about family planning, different methods and proper use of contraception, prevention and management of malaria in pregnancy and interventions for smoking cessation during pregnancy for improving birth outcomes. At the referral level, procedures should be available for those seeking long term contraception such as intra-uterine device, tubal ligation, surgical contraception as well as long acting reversible contraceptives (implants). Prevention and management of STIs; including prevention of mother to child transmission (PMTCT) of HIV and syphilis by the use provisions such as HIV test kits, ARV drugs and cotrimoxazole should be practiced in all areas with high prevalence of these diseases.

#### 2. Nutrition support care package for mothers

This package consists of key nutrition interventions, including periconceptual folic acid supplementation, iron/folic acid during pregnancy, and calcium supplementation in pregnant women with low/inadequate calcium intake. These interventions can be both promoted and delivered at the community level. Supplementation can also be administered via outpatient programs. The women should also be counselled to make changes in their diets to improve their health status. Folic acid fortification and/or supplementation to prevent NTDs must be advocated and use of folic acid fortified staple food can be advised to all those who are pregnant or plan to conceive in the future. Iron and folic acid supplementation during pregnancy should also be advised to those women who show signs of anaemia. Calcium supplementation in pregnant women with low/inadequate calcium intake has also been shown to be beneficial and must be advocated to those in whom it is justified.

#### 3. Antenatal care package

Antenatal care can be delivered at the primary and outpatient level. The package must include birth preparedness at the nearest health care centre and availability of trained birth attendants and sterile surgical equipment, risk detection by the use of fetal stethoscopes and ultrasound, and counselling and health promotion at all levels of health care in an integrated approach. Equipment such as fetal stethoscope‚ scale‚ sphygmomanometer‚ haemoglobin-meter must be available at primary health care settings. Detection, treatment and management of maternal risks factors are included in this package and that includes management of malaria, HIV and STI infections. The early detection should result in prompt treatment at the primary of referral health care centres by a certified medical professional. Anaemia, pre-eclampsia and nutritional deficiencies in mothers can be screened at outpatient visits. However, at the community level, mothers can also be counselled on the benefits of breastfeeding and newborn care and health care seeking. It is also important to ensure that the CHWs have a generally good rapport to improve compliance and also to be able to detect any risk earlier through regular scheduled home visits. Few other interventions such as tetanus immunization during pregnancy can also be delivered at the level of the community or via outpatient/outreach programs. It is important to advocate the delivery of the vaccine for the prevention of any complication arising from lack of immunization.

#### 4. Package for high risk pregnancy

Routine antenatal care can be expanded by adding few important interventions for women who are at high risk of pregnancy. These interventions allow management of pregnancy-induced hypertension (PIH), pre-eclampsia and eclampsia. These interventions are more appropriate at the tertiary care level after thorough clinical and laboratory assessment, but could be adapted for implementation at lower levels of the health care system. The availability of Uterotonic (Oxytocin and/or Misoprostol), partograph for the management of pre mature rupture of membranes should be ensured to avoid any loss of life arising due to such complications. Stocks of Calcium, Low dose Aspirin, Methyldopa, hydralazine, nifedipine, Magnesium Sulphate (Injection) should be available at the pharmacy for those who require treatment in primary as well as referral levels of health care to ensure timely delivery of these crucial interventions and reduction for the need to travel long distances to acquire these services. Health care professionals should also be trained to plan and manage high risk pregnancy as well as respond to any emergency that may arise.

#### 5. Childbirth care package

Childbirth care package is crucial to the survival of both the mother and the neonate in the critical period of delivery and post-partum. External cephalic version must be employed by trained health care professionals to reduce malpresentation, induction of labor for prolonged pregnancy, active management of third stage of labor are few of the interventions that can be delivered at tertiary level. Adequate sterilization of surgical environment must be ensured for caesarean section and instrumental delivery by health care professionals. These interventions require tertiary level care. These interventions should be considered essential components of any childbirth care package and includes training of skilled birth attendance (doctors, nurses, midwives), and traditional birth attendant (TBAs)for clean delivery and referral. Another implementation strategy could be the provision of birth kits to the TBAs will ensure access to this facility to those residing in remote areas. This will reduce the mortality incidence that arises from delay in the provision of emergency medical aid during childbirth.

#### 6. Essential newborn care package

In the postnatal period, interventions such as breastfeeding, thermal care, kangaroo mother care, skin and cord care can be provided by CHWs. These interventions can be integrated into another important postpartum intervention, namely, postnatal visits. At the level of the tertiary care center, postnatal care, particularly for low birth weight infants and newborns at-risk.

#### 7. Child health care package

Malnutrition and infections carry the greatest brunt of deaths in children less than 5 years of age. Therefore, child health support includes nutritional support and infection management interventions. Exclusive breastfeeding, complementary feeding and vitamin A supplementation are some of the nutritional interventions that can be delivered at community level by developing support groups for women for education and empowerment, and training community health workers. Case management of childhood illness, such as malaria, pneumonia and diarrhea can be performed in the outpatient setting, whereas management of HIV and STIs should preferably be done at a tertiary care center. Immunization for EPI, pneumococcal vaccine and Rota virus vaccine are some of the other interventions that can be delivered at primary or tertiary levels.

### Implementation strategies

**Figure 2:**
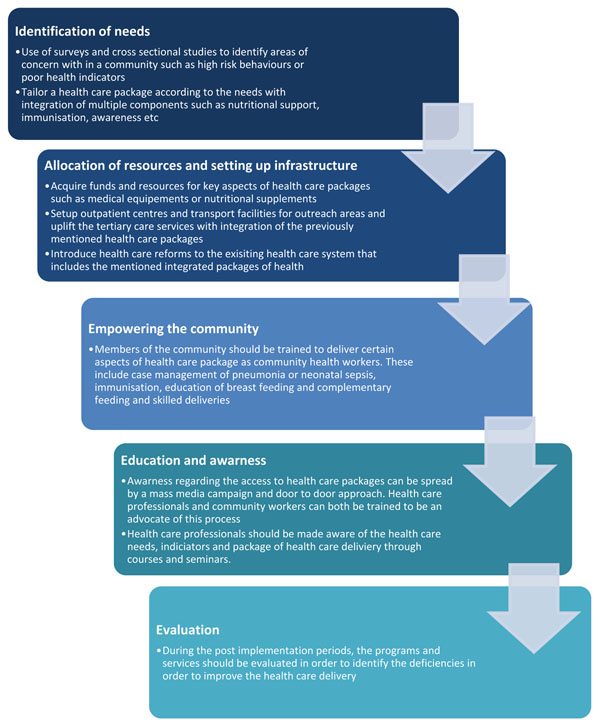
Implementation strategies for delivery of the proposed health care package

Implementation of the health care packages discussed earlier is the most crucial and important task at hand. In order to better understand the implementation strategies we have to take into account different hurdles that have halted the provision of health care. It is vital to understand the fact that these packages are central for low income countries where maternal and child health indicators are still not up to standard. Some of the known reasons for the poor health indicators in these areas include low levels of education, poor awareness regarding health care services, lack of infrastructure to support health care, deprived sanitation and hygienic practices, poverty, dearth of access to communities as a result of armed/political conflicts and poor acceptance of health care services as a result of cultural and social practices and beliefs. The implementation strategies thus have to tackle with these issues in order to successfully provide the health care package and improve reproductive, maternal, neonatal and child care outcomes. We have devised and highlighted the following strategies that can be useful in such scenarios (see figure 2), however, it is important to understand that these strategies are sequential to a large extent but some of the steps need not follow the others and can be implemented concurrently.

#### Allocation of funds and resources

While we have outlined the interventions needed to improve targeted issues, not every part of the world has the same problems. It is thus essential to identify the needs and concerns of a particular area and then allocate resources and funds accordingly. For instance a community may have a high maternal mortality rate but a low prevalence of HIV. Here funds should be redirected towards antenatal and intrapartum care rather than on packages focusing on management of HIV. This allows a balance and focused approach and improves health care as needed by the community. Identification of needs can be accomplished by simple cross sectional studies, surveys and assessment of the health care records. Community members can be empowered and trained to carry out these tasks as well. The collected data can then be used to gather funds and resources from government and non-government organizations for improving health care delivery.

#### Supporting infrastructure

It is necessary to provide a sustaining infrastructure to improve RMNCH outcomes. This requires input from multiple levels especially the NGOs and local authorities. Allocation of funds to important areas of health care that are responsible for poor outcomes is a necessary step. Provision of primary health care facilities and availability of trained medical staffs should be emphasized upon. In communities, where the access to health facility is an issue, transport facilities should be provided. . By supporting infrastructure we can strengthen the facility care.

#### Community health workers

Community health workers and TBAs can play a significant role in improving maternal and newborn health care outcomes. Empowering and training people from within the community and allowing them to address health care issues can overcome cultural and social practices and beliefs that do not allow access to health care. CHWs can not only provide awareness and education but also play a significant role in providing case management of maternal and newborn infections, immunizations, identification and management of high risk pregnancies and provision of primary and referral services. CHWs can provide a much needed bridge between communities that do not have an access to health care facilities and health facilities.

#### Education and awareness

While educating the community about health care needs has always been emphasized upon, it is also important to educate health care professionals on the needs and health care issues of the community. Through courses, seminars and conferences health care professionals should be informed about the important health care issues of the community. They should also be encouraged to participate in discussions to come up with ideas and solutions for the problems at hand. This will allow the health care providers of the community to better understand the problems and hence allow them to support the people in a much better way. Awareness regarding packages of health care can also be created at community level by posters and bill boards in heavily frequented streets and hospitals. The intervention can also be implemented by government or NGO funded advertisement campaigns on radio or television during prime time to ensure maximum reach. At the same time, the role of health care providers is imperative in the education and awareness of the general population. Health care providers can carry out a longitudinal process of educating the population at primary, secondary as well as tertiary level of health care. Target groups for specific populations could be made and their specific issues could be addressed by the government or NGOs such as providing awareness of antenatal health care interventions to women of the reproductive age group in the community. Such measures would ensure the maximum reach and awareness and enable effective education of the general population.

#### Evaluation

Once these health care packages are provided to people, the impact should be evaluated. This would help in identifying areas of improvement and thus overcome the deficiencies of implementation strategies. Small pilot programs can also be conducted to better understand the needs and focus health care provision according to the requirements. It is important to address the specific needs of the community. Many a times, interventions have to be delivered in a way most suitable to the community. At the same time, factors that are hindering the delivery of the interventions need to be addressed e.g. the provision of safe abortions should be advocated particularly in community of sex workers. Hence implementation should match the needs of the community.

## Conclusion

This review was designed to serve as a comprehensive and thorough document of essential RMNCH interventions which could be used by both clinicians and policy makers. The implementation of these interventions with suggested strategies is a combined responsibility of both the public and private health sector and requires a concerted and well planned effort. Working in synergy of both these group including the NGOs contributing to the health sector would ensure maximum implementation within a relatively short frame of time. These interventions should be integrated in health policies and programs as part of a continuum of care approach. The integration of these essential maternal and newborn health strategies has the potential to improve efficiency, save scarce resources, and ultimately improve maternal and newborn outcomes. Implementation of these interventions will help reduce the mortality rates in areas where maternal, neonatal and child mortality rates remain high due to lack of specific interventions. This also requires health system to adapt to these needs and optimize the benefits of these interventions to the community by introducing policies which help in the implementation of these interventions. Funds need to be allocated to obtain the commodities which are essential for the delivery of these interventions. This approach is critical to achieving MDGs 4 and 5 – ensuring that we not only save lives but also to address overall health and well-being.

## Reviewer reports

The reviewer reports for this article can be found in additional file 1.

## Competing interests

We do not have any financial or non-financial competing interests for this review.

## Peer review

The reviewer reports for this article can be found in Additional File 1.

## Supplementary Material

Additional file 1 Reviewer reports. Click here for file

## Declarations

This article has been published as part of *Reproductive Health* Volume 11 Supplement 1, 2014: Essential intervention for maternal, newborn and child health. The full contents of the supplement are available online at http://www.reproductive-health-journal.com/supplements/11/S1. Publication charges for this collection were funded by the Partnership for Maternal, Newborn & Child Health (PMNCH).
