# Supplementary material for: Essential interventions: implementation strategies and proposed packages of care
Source: Reprod Health. 2014 Aug 21;11(Suppl 1):S5. doi: 10.1186/1742-4755-11-S1-S5 (PMC4145859; doi:10.1186/1742-4755-11-S1-S5)
Supplement: Additional file 1 — Reviewer reports. [file 1742-4755-11-S1-S5-S1.pdf]

Referee's comments to the authors– this sheet WILL be seen by the author(s) and published with the article

Title	**Essential Interventions: implementation strategies and proposed packages of care**
Author(s)	Zohra S Lassi, Rohail Kumar, Tarab Mansoor, Rehana A Salam, Jai K Das, Zulfiqar A Bhutta
Referee's name	Diego Bassani

When assessing the work, please consider the following points, where applicable:

1. Is the question posed by the authors new and well defined?
2. Are the methods appropriate and well described, and are sufficient details provided to replicate the work?
3. Are the data sound and well controlled?
4. Does the manuscript adhere to the relevant standards for reporting and data deposition?
5. Are the discussion and conclusions well balanced and adequately supported by the data?
6. Do the title and abstract accurately convey what has been found?
7. Is the writing acceptable?

Please make your report as constructive and detailed as possible in your comments so that authors have the opportunity to overcome any serious deficiencies that you find and please also divide your comments into the following categories:

- Major Compulsory Revisions (which the author must respond to before a decision on publication can be reached)
- Minor Essential Revisions (such as missing labels on figures, or the wrong use of a term, which the author can be trusted to correct)
- Discretionary Revisions (which are recommendations for improvement but which the author can choose to ignore)

Where possible please supply references to substantiate your comments.

When referring to the manuscript please provide specific page and paragraph citations where appropriate.

General comments: The paper addresses an important issue and makes an interesting summary of implementation strategies and packages of care for essential interventions in RMNCH.

Major compulsory revisions: None of the recommendations/suggestions are referenced and this is a major issue in the manuscript.

Minor essential revisions: There are a series of grammar issue that deserve attention of the authors. A full revision of the text should be undertaken. On page 4, the comment of folic acid reducing anemia should mention that it is delivered with iron and this is he reason anemia is reduced unless the authors are talking about folate anemia.

The paper refers to the 'series' it seems to be part of but it is not at all clear what it means when read in isolation.

On Page 9 the authors talk about preventing unhealthy behaviors and emotions and I think this should be properly revised and clarified – can you prevent unhealthy emotions?

(continue on the next sheet)

Referee's comments to the authors– this sheet WILL be seen by the author(s) and published with the article

Title	**Essential Interventions: implementation strategies and proposed packages of care**
Author(s)	Zohra S Lassi, Rohail Kumar, Tarab Mansoor, Rehana A Salam, Jai K Das, Zulfiqar A Bhutta
Referee's name	M Jeeva Sankar

When assessing the work, please consider the following points, where applicable:

1. Is the question posed by the authors new and well defined?	**Not applicable**
2. Are the methods appropriate and well described, and are sufficient details provided to replicate the work?	**Not applicable**
3. Are the data sound and well controlled?	**Not applicable**
4. Does the manuscript adhere to the relevant standards for reporting and data deposition?	**Not applicable**
5. Are the discussion and conclusions well balanced and adequately supported by the data?	**Not applicable**
6. Do the title and abstract accurately convey what has been found?	**Yes**
7. Is the writing acceptable?	**Yes (to a large extent – see comments below)**

Please make your report as constructive and detailed as possible in your comments so that authors have the opportunity to overcome any serious deficiencies that you find and please also divide your comments into the following categories:

- Major Compulsory Revisions (which the author must respond to before a decision on publication can be reached)
- Minor Essential Revisions (such as missing labels on figures, or the wrong use of a term, which the author can be trusted to correct)
- Discretionary Revisions (which are recommendations for improvement but which the author can choose to ignore)

Where possible please supply references to substantiate your comments.

When referring to the manuscript please provide specific page and paragraph citations where appropriate.

General comments:

The paper enlists the essential interventions for improving maternal, newborn and child health, discusses how they can be combined in the form of packages, and outlines the possible implementation strategies of them. Prima facie, it looks like an independent review paper but lacks the usual rigor required for one – general statements, no sourcing of data, and no references/evidence back-up for the list of interventions mean that the paper is not a free-standing one. However, I understand that this paper is a part of the supplement on "Essential Interventions for Maternal, Newborn and Child Health" (fifth paper in the supplement!). It is quite possible that the first four articles might have already addressed many of these issues. Without knowing the contents of other papers, it is therefore really difficult to comment on this paper.

Major compulsory revisions:

None

(continue on the next sheet)

Minor essential revisions:

1. The section ‘Summary of essential interventions’ would be superfluous, if the other papers in the series were to enumerate the same. If it is still needed to set the stage for further discussion, it can be trimmed significantly. Currently, this section constitutes more than half of the entire paper. It would be preferable to have 1-2 tables that enlist the interventions instead of the text. The tables would enable the readers to have a snapshot of the interventions that are effective at different levels.
2. There is also a need to have references for each of the listed interventions - it would help the reader to examine the source of the evidence. This is particularly important for some interventions that are novel and/or not being practiced routinely (e.g. use of antiplatelet agents in *all* women with gestational hypertension).
3. For some of the listed interventions, the evidence for efficacy mentioned in the paper is not up-to-date. For example, the paper mentions that *“antiseptic chlorhexidine was associated with lower risk of bacterial colonization with S. aureus, Streptococci and E. coli…”* but does not elaborate on the effect of the intervention on the risk of omphalitis or neonatal mortality.
4. As the authors rightly pointed out, the continuum of care approach has two major components – maternal, newborn, child health (MNCH) continuum of care *and* the household to hospital continuum of care (HHCC). The packages of care proposed by the authors do address the first component but not necessarily the other one. Figure 1 does indicate the appropriate level of care for each intervention but does not depict it in the typical ‘continuum of care’ approach. I do understand the difficulty in linking the packages to both the level of care and the time period. But such an effort would be of great help to key stakeholders.
5. The implementation strategies outlined in the paper gives an impression that the steps are sequential (first followed by second, and so on). This is of course true to a large extent but some of the steps need not follow the others – they can happen concurrently. For example, empowering the community or education and awareness can happen even before/during the setting-up of infrastructure.
   Also, the process should be a cyclical one with the evaluation (last step) leading to identification of needs (first step) and thereby initiating the next cycle. Some of the components - like community empowerment - can act at different stages of the cycle.
6. It would help if the paper enlists the benefits and potential impact of the interventions, and the health system requirements (policy, service delivery, supplies and commodities, indicators, etc.) that need to be in place to deliver the interventions at various levels.

Discretionary revisions:

1. The discussion on implementation strategies does not address a few important points like identifying and addressing bottlenecks, specific role of different cadres of health care providers and other stakeholders, etc. The authors, with their vast experience in these issues, can really educate the readers regarding these issues.

Other comments:

In general, the paper is well written. It would, however, be difficult to comment if it has achieved its overarching objectives in the context of the supplement without knowing the content of the other papers in the series.

Supplement Editor's comments

Further to the comments of the reviewers please take in account these recommendations.
The abstract should be rewritten. It should provide information about the major findings and conclusions of the paper instead of saying “This paper has summarized the essential interventions as per the level of health care and in the form of care packages which could then be delivered across the continuum of care. This paper has also discussed the implementation strategies for employing these interventions at scale..” Please provide the information about the findings and conclusions.
Please give a better description of the objective of this paper.
Both reviewers raised concerns about the English editing. One suggestion is to use the service of Edanz to improve this.
http://www.edanzediting.com/

Referee's comments to the authors– this sheet WILL be seen by the author(s) and published with the article

Title	**Essential Interventions: implementation strategies and proposed packages of care**
Author(s)	Zohra S Lassi, Rohail Kumar, Tarab Mansoor, Rehana A Salam, Jai K Das, Zulfiqar A Bhutta
Referee's name	Diego Bassani

When assessing the work, please consider the following points, where applicable:

1. Is the question posed by the authors new and well defined?
2. Are the methods appropriate and well described, and are sufficient details provided to replicate the work?
3. Are the data sound and well controlled?
4. Does the manuscript adhere to the relevant standards for reporting and data deposition?
5. Are the discussion and conclusions well balanced and adequately supported by the data?
6. Do the title and abstract accurately convey what has been found?
7. Is the writing acceptable?

Please make your report as constructive and detailed as possible in your comments so that authors have the opportunity to overcome any serious deficiencies that you find and please also divide your comments into the following categories:

- Major Compulsory Revisions (which the author must respond to before a decision on publication can be reached)
- Minor Essential Revisions (such as missing labels on figures, or the wrong use of a term, which the author can be trusted to correct)
- Discretionary Revisions (which are recommendations for improvement but which the author can choose to ignore)

Where possible please supply references to substantiate your comments.

When referring to the manuscript please provide specific page and paragraph citations where appropriate.

General comments: The paper addresses an important issue and makes an interesting summary of implementation strategies and packages of care for essential interventions in RMNCH.

Major compulsory revisions: None of the recommendations/suggestions are referenced and this is a major issue in the manuscript.
We have now provided references to the studies from which the evidence has been generated

Minor essential revisions: There are a series of grammar issue that deserve attention of the authors. A full revision of the text should be undertaken. On page 4, the comment of folic acid reducing anemia should mention that it is delivered with iron and this is he reason anemia is reduced unless the authors are talking about folate anemia. Corrected.

The paper refers to the 'series' it seems to be part of but it is not at all clear what it means when read in isolation. We now provided all the references so it would be easy to follow.

On Page 9 the authors talk about preventing unhealthy behaviors and emotions and I think this should be properly revised and clarified – can you prevent unhealthy emotions? Corrected.

(continue on the next sheet)

Referee's comments to the authors– this sheet WILL be seen by the author(s) and published with the article

Title	**Essential Interventions: implementation strategies and proposed packages of care**
Author(s)	Zohra S Lassi, Rohail Kumar, Tarab Mansoor, Rehana A Salam, Jai K Das, Zulfiqar A Bhutta
Referee's name	M Jeeva Sankar

When assessing the work, please consider the following points, where applicable:

8. Is the question posed by the authors new and well defined?	**Not applicable**
9. Are the methods appropriate and well described, and are sufficient details provided to replicate the work?	**Not applicable**
10. Are the data sound and well controlled?	**Not applicable**
11. Does the manuscript adhere to the relevant standards for reporting and data deposition?	**Not applicable**
12. Are the discussion and conclusions well balanced and adequately supported by the data?	**Not applicable**
13. Do the title and abstract accurately convey what has been found?	**Yes**
14. Is the writing acceptable?	**Yes (to a large extent – see comments below)**

Please make your report as constructive and detailed as possible in your comments so that authors have the opportunity to overcome any serious deficiencies that you find and please also divide your comments into the following categories:

- Major Compulsory Revisions (which the author must respond to before a decision on publication can be reached)
- Minor Essential Revisions (such as missing labels on figures, or the wrong use of a term, which the author can be trusted to correct)
- Discretionary Revisions (which are recommendations for improvement but which the author can choose to ignore)

Where possible please supply references to substantiate your comments.

When referring to the manuscript please provide specific page and paragraph citations where appropriate.

General comments:

The paper enlists the essential interventions for improving maternal, newborn and child health, discusses how they can be combined in the form of packages, and outlines the possible implementation strategies of them. Prima facie, it looks like an independent review paper but lacks the usual rigor required for one – general statements, no sourcing of data, and no references/evidence back-up for the list of interventions mean that the paper is not a free-standing one. However, I understand that this paper is a part of the supplement on "Essential Interventions for Maternal, Newborn and Child Health" (fifth paper in the supplement!). It is quite possible that the first four articles might have already addressed many of these issues. Without knowing the contents of other papers, it is therefore really difficult to comment on this paper.

We have now provided references to the studies from which the evidence has been generated

Major compulsory revisions:

None

(continue on the next sheet)

Minor essential revisions:

7. The section 'Summary of essential interventions' would be superfluous, if the other papers in the series were to enumerate the same. If it is still needed to set the stage for further discussion, it can be trimmed significantly. Currently, this section constitutes more than half of the entire paper. It would be preferable to have 1-2 tables that enlist the interventions instead of the text. The tables would enable the readers to have a snapshot of the interventions that are effective at different levels.
   Added a table.
8. There is also a need to have references for each of the listed interventions - it would help the reader to examine the source of the evidence. This is particularly important for some interventions that are novel and/or not being practiced routinely (e.g. use of antiplatelet agents in *all* women with gestational hypertension). Added
9. For some of the listed interventions, the evidence for efficacy mentioned in the paper is not up-to-date. For example, the paper mentions that *"antiseptic chlorhexidine was associated with lower risk of bacterial colonization with S. aureus, Streptococci and E. coli…"* but does not elaborate on the effect of the intervention on the risk of omphalitis or neonatal mortality. Added
10. As the authors rightly pointed out, the continuum of care approach has two major components – maternal, newborn, child health (MNCH) continuum of care *and* the household to hospital continuum of care (HHCC). The packages of care proposed by the authors do address the first component but not necessarily the other one. Figure 1 does indicate the appropriate level of care for each intervention but does not depict it in the typical 'continuum of care' approach. I do understand the difficulty in linking the packages to both the level of care and the time period. But such an effort would be of great help to key stakeholders. Added under the heading of proposed packages of care
11. The implementation strategies outlined in the paper gives an impression that the steps are sequential (first followed by second, and so on). This is of course true to a large extent but some of the steps need not follow the others – they can happen concurrently. For example, empowering the community or education and awareness can happen even before/during the setting-up of infrastructure.
    Also, the process should be a cyclical one with the evaluation (last step) leading to identification of needs (first step) and thereby initiating the next cycle. Some of the components - like community empowerment - can act at different stages of the cycle. Well we logically ordered them but now have specified them explicitly in implementation strategies : 1st para and last sentence.
12. It would help if the paper enlists the benefits and potential impact of the interventions, and the health system requirements (policy, service delivery, supplies and commodities, indicators, etc.) that need to be in place to deliver the interventions at various levels. Added in table 1.

Discretionary revisions:

2. The discussion on implementation strategies does not address a few important points like identifying and addressing bottlenecks, specific role of different cadres of health care providers and other stakeholders, etc. The authors, with their vast experience in these issues, can really educate the readers regarding these issues. Added in table 1.

Other comments:

In general, the paper is well written. It would, however, be difficult to comment if it has achieved its overarching objectives in the context of the supplement without knowing the content of the other papers in the series.

Supplement Editor's comments

Further to the comments of the reviewers please take in account these recommendations.
The abstract should be rewritten. It should provide information about the major findings and conclusions of the paper instead of saying "This paper has summarized the essential interventions as per the level of health care and in the form of care packages which could then be delivered across the continuum of care. This paper has also discussed the implementation strategies for employing these interventions at scale.." Please provide the information about the findings and conclusions.
Please give a better description of the objective of this paper. Abstract is now re written
Both reviewers raised concerns about the English editing. One suggestion is to use the service of Edanz to improve this.
http://www.edanzediting.com/
